# Genome Wide Analysis of Amino Acid Transporter Superfamily in *Solanum lycopersicum*

**DOI:** 10.3390/plants10020289

**Published:** 2021-02-03

**Authors:** Fatima Omari Alzahrani

**Affiliations:** Department of Biology, Faculty of Science, Albaha University; Albaha 65527, Albaha Province, Saudi Arabia; drfatimaomari@gmail.com

**Keywords:** tomato, phylogenetic analysis, expression, evolution

## Abstract

Amino acid transporters (AATs) are integral membrane proteins and have several functions, including transporting amino acids across cellular membranes. They are critical for plant growth and development. This study comprehensively identified AAT-encoding genes in tomato (*Solanum lycopersicum*), which is an important vegetable crop and serves as a model for fleshy fruit development. In this study, 88 genes were identified in the *S. lycopersicum* genome and grouped into 12 subfamilies, based on previously identified AATs in *Arabidopsis*, rice (*Oryza sativa*), and potato (*Solanum tuberosum*) plants. Chromosomal localization revealed that *S. lycopersicum* AAT (*SlAAT*) genes are distributed on the 12 *S. lycopersicum* chromosomes. Segmental duplication events contribute mainly to the expansion of *SlAAT* genes and about 32% (29 genes) of *SlAAT* genes were found to originate from this type of event. Expression profiles of *SlAAT* genes in various tissues of *S. lycopersicum* using RNA sequencing data from the Tomato Functional Genomics Database showed that *SlAAT* genes exhibited tissue-specific expression patterns. Comprehensive data generated in this study will provide a platform for further studies on the SlAAT gene family and will facilitate the functional characterization of *SlAAT* genes.

## 1. Introduction

Tomato (*Solanum lycopersicum*) has great global commercial importance owing to its nutritional value and serves as a model for fleshy fruit development and as a reference species for plants in the *Solanaceae* family. Several genome-wide studies have identified 63, 87, 23, 189, and 72 genes encoding amino acid transporters (AATs) in *Arabidopsis thaliana*, *Oryza sativa*, *Selaginella*, *Glycine max*, and *Solanum tuberosum*, respectively [1,2,3,4,5]. Although AATs have been studied in several plant species, there is limited information available about AATs in *S. lycopersicum*. 

AATs are integral membrane proteins, which mediate the nitrogen allocation between source and sink [6] in plants. The phloem sap is rich in amino acids and they are responsible for procuring the organic [2]; nitrogen that is necessary for plant growth and development [7]. Furthermore, AATs play fundamental roles in plant physiological processes such as defense against pathogens and resistance to abiotic stresses. 

AATs are divided into two families; the first is amino acid/auxin permease (AAAP), which is in turn sub-grouped into eight subfamilies, including amino acid permease (AAP), lysine/histidine transporter (LHT), γ-aminobutyric acid (GABA) transporter (GAT), proline transporter (ProT), like-auxin influx carriers (LAX), aromatic and neutral amino acid transporter (ANT), vesicular aminergic-associated transporter (VAAT), and amino acid transporter-like proteins (ATL) [8]. The second family comprises of amino acid-polyamine-choline (APC) transporters, which in turn is sub-grouped into four subfamilies, including amino acid/choline transporters (ACT), tyrosine-specific transporter (TTP), cationic amino acid transporter (CAT), and polyamine H^+^ co-transporters (PHS) [9]. In addition, a new subfamily has been added to the AAT superfamily called Usually Multiple Amino Acids Move In and Out Transporters (UMAMIT) [10].

A few studies have functionally characterized the members of the AAT superfamily in plants. Eight AAPs have been identified in *Arabidopsis*, which are localized in the plasma membrane and involved in the H^+^-coupled amino acid uptake system [11]. AAP1 and AAP2 in *Arabidopsis*, the first two AATs to be characterized, have different substrate specificity with high expression in siliques of *Arabidopsis*, suggesting their involvement in supplying the seeds with organic nitrogen [12]. AAP3, AAP4, and AAP5 were later isolated from *Arabidopsis* with broad substrate specificities and AAP5 was found to participate in amino acid transport in the developing embryo [13]. *Arabidopsis thaliana* AAP7 (AtAAP7) is still not functionally characterized. AtAAP6 and AtAAP8 are involved in high-affinity amino acid transport, since AAP8 imports organic nitrogen into developing seeds and AAP6 is expressed in xylem, which has low concentrations of amino acids [14]. Similar to *Arabidopsis*, eight members of AAPs have been identified in *S. tuberosum* [5], and 19 have been identified in *O. sativa* [1]. AAPs in other plant species have also been functionally characterized. For example, AAP1 is expressed in the leaves and is involved in the long-distance transport of amino acids in *S. tuberosum* [15]. Three primases, including VfAAP1, VfAAP3, and VfAAP4 were isolated from *Vicia faba* L. and have been shown to transport a broad range of amino acids [16].

Ten LHTs (AtLHT1–10) have been identified in *Arabidopsis*. They were found to be localized in the plasma membrane and were found to import organic nitrogen into the roots, mesophyll cells [17], and the cells of reproductive floral tissue [18]. AtLHT1 together with AtAAP5 are involved in the uptake of neutral, acidic, and basic amino acids from the soil when amino acid levels in the nutrient solutions (or soil) are low [19,20].

Unlike AAPs and LHTs, the only proteinogenic amino acid transported by ProTs is proline [21]. However, studies have shown that ProTs are responsible for transporting glycine betaine and GABA [22]. Three ProTs (AtProT1, AtProT2, AtProT3) were identified in *Arabidopsis* and it was shown that despite the similar localization of the three transporters in the plasma membrane and similar affinity to glycine betaine, each transporter has a different role in *Arabidopsis* [22]. For example, AtProT1 is highly expressed in the phloem, suggesting its involvement in long-distance transport of compatible solutes. AtProT2 is active in the roots, while AtProT3 is active in the epidermal cells of the leaves [22].

ProT1 in *S. lycopersicum* is a general transporter for compatible solutes [23] and transports proline to roots even under salt-stress conditions [24]. Regarding GATs in *Arabidopsis*, it was revealed that AtGAT1 has a high affinity to GABA and is localized in the plasma membrane [25]. In addition, it is highly expressed in flowers under elevated GABA conditions, such as wounding and senescence [25].

*Aux1* mutant *Arabidopsis* plants show defects in the root gravitropic response, and *AtAUX1* is expressed in the columella, lateral root cap, epidermis, and stele tissues of the primary root [26]. AtAUX1 together with AtPIN2 (auxin exporter) regulate root gravitropism [27], and AtAUX1 promotes lateral root formation and phyllotactic pattern in *Arabidopsis* [28]. In *Arabidopsis*, the *AUX1* gene belongs to a small family consisting of four members, including *AUX1* and three *LAX* genes (*LAX1*, *2*, and *3*) [29], while there are five AUX/LAX transporters in *O. sativa* [30]. Regarding ANT transporters, only AtANT1 was characterized in *Arabidopsis*, which transports arginine, indole-3-acetic acid, and 2,4-dichlorophenoxyacetic acid [31]. Of CAT transporters, nine members have been functionally characterized in *Arabidopsis* with varying affinities and functions [32]. For example, AtCAT5 is a high-affinity basic amino acid transporter and is involved in reuptake of the leaking amino acids at the leaf margin, while AtCAT2 is involved in long-sought vacuolar amino acid transport [32]. Recently, six CAT members were identified and characterized in *Camellia sinensis* and their expression was revealed to be sensitive to abiotic stress [33]. In *Arabidopsis*, a bidirectional amino acid transporter (BAT), which performs both exporting and importing activities, was reported to exhibit transport activity for alanine, arginine, glutamate, and lysine [34].

Since the functions of *S. lycopersicum* AATs are not fully studied, we aimed to elucidate the entire members of AAT gene superfamily in *S. lycopersicum*. In this study, a genome-wide identification and phylogenetic analysis of *S. lycopersicum* AAT (*SlAAT*) genes were performed for AAT superfamily classification and to explore the evolution of this gene superfamily. In addition, the features of the exon-intron structures, patterns of the conserved motifs, and duplication events, including tandem and segmental duplications within the tomato genome that likely contribute to the expansion of the SlAAT superfamily were explored. The resulting data will be useful in studies on the biological functions of each gene in the SlAAT superfamily.

## 2. Results

### 2.1. Identification of SlAATs in the S. lycopersicum Genome

**Figure 1:**
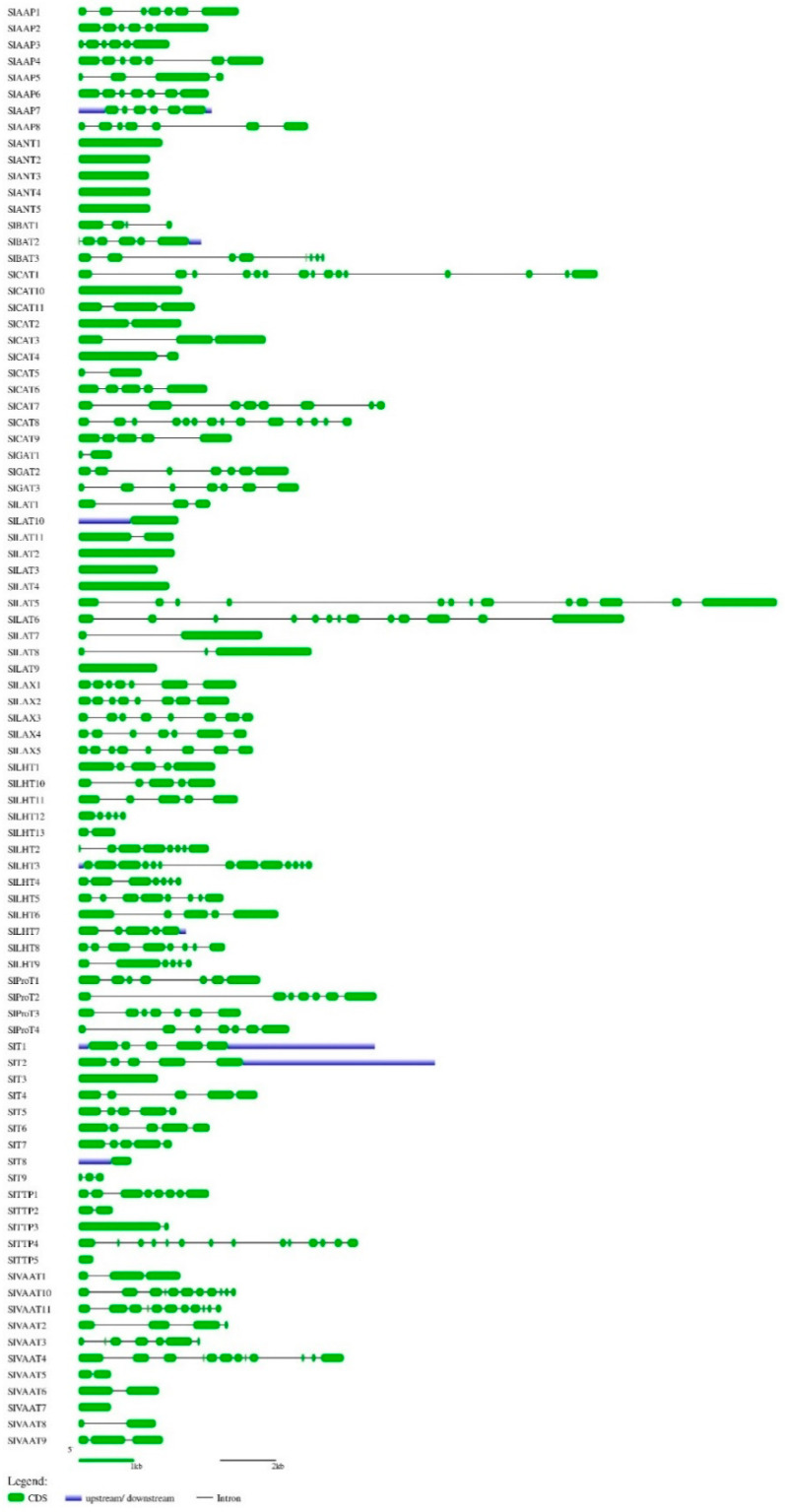
Exon-intron structures of the identified *SlAAT* genes. The graphic representation of the optimized gene model displayed using Gene Structure Display Server (GSDS) (for interpretation of the references to colors in this figure, the reader is referred to the web version of this article). Green boxes represent exons. The black solid lines connecting two exons represent introns, and the blue boxes represent upstream/downstream region of *SlAAT* genes. AAP, amino acid permease; ANT, aromatic and neutral amino acid transporter; ATL, amino acid transporter-like proteins; BAT, bidirectional amino acid transporter; CAT, cationic amino acid transporter; GAT, γ-aminobutyric acid transporter; LAT, L-type amino acid transporter; LAX, like-auxin influx carrier; LHT, lysine/histidine transporter; ProT, proline transporter; TTP, tyrosine-specific transporter; VAAT, vesicular aminergic-associated transporter.

Using ATTs of *Arabidopsis thaliana*, *O. sativa*, and *S. tuberosum* as queries in BLAST search at SGN with ‘amino acid transporter’ and ‘amino acid permease’ as keywords, we identified 88 members of SlAATs. All the retrieved protein sequences of SlAATs were subjected to InterProScan (http://www.ebi.ac.uk/Tools/InterProScan/ (accessed on 31 January 2021)), and all the candidate proteins were found to contain AAT domain(s). Information about locus identity number of *SlAATs* assigned by SGN (Solanaceae Genomics Network, http://solgenomics.net/SOL (accessed on 31 January 2021)), the given nomenclature of *S. lycopersicum* AATs, number of intron(s) in the *SlAAT* genes, length of the ORF for *SlAATs*, protein characterization of SlAATs (amino acid length, MW, pI), and genomic location are presented in Table 1. The intron number ranged from 1 to 13 and the length of the ORF for *SlAATs* ranged from 267 to 3376 bp. It was observed that each subfamily member shared a nearly similar gene structure and intron numbers. For example, members of ProT subfamily contained six introns and an ORF length of 1473–1828 bps. The intron-exon regions in each genomic sequence of *SlAAT* are illustrated in Figure 1. 

**Figure 2:**
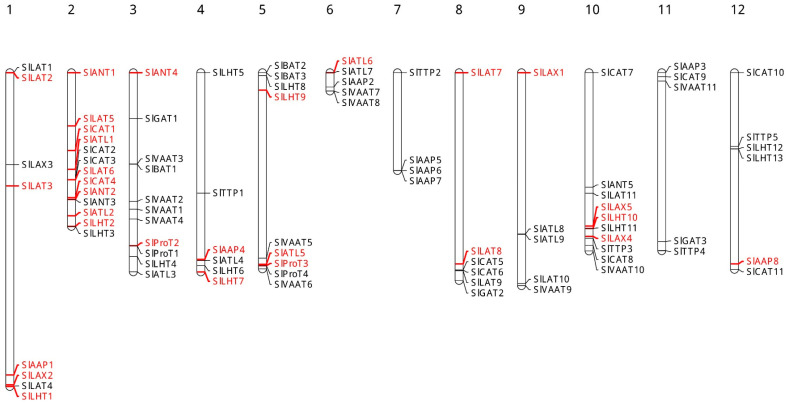
Chromosomal localization of *SlAAT* genes. Respective chromosome numbers are indicated at the top of each bar. Gene duplication events of *SlAAT* genes marked in red. (For interpretation of the references to color in this figure, the reader is referred to the web version of this article.) AAP, amino acid permease; ANT, aromatic and neutral amino acid transporter; ATL, amino acid transporter-like proteins; BAT, bidirectional amino acid transporter; CAT, cationic amino acid transporter; GAT, γ-aminobutyric acid transporter; LAT, L-type amino acid transporter; LAX, like-auxin influx carriers; LHT, lysine/histidine transporter; ProT, proline transporter; TTP, tyrosine-specific transporter; VAAT, vesicular aminergic-associated transporter.

The number of transmembrane regions (Table 1), which were predicted by the TMHMM Server, were found to range from 2 to 18 and some members of each subfamily of SlAATS shared a similar number of transmembrane regions. Using the information available in the SGN about SlAATs, it was determined that *SlAAT* genes are distributed in all the 12 chromosomes in *S. lycopersicum* (Figure 2). An uneven distribution of *SlAAT* genes on the 12 chromosomes was observed; for example, chromosome 2 contained 13 *SlAAT* genes, while chromosome 7 contains four *SlAAT* genes. The gene duplication data obtained from PGDD (Plant Genome Duplication Database, http://chibba.agtec.uga.edu/duplication (accessed on 31 January 2021)) (*S. lycopersicum* vs. *S. lycopersicum)* revealed that 29 *SlAAT* genes originate from the duplication events and there are 16 gene pairs of *SlAATs* (Table 2). Examining the 16 gene pairs according to the criterion of tandem duplication, all the duplication events were characterized to be segmental duplication. Thus, the divergence time ranged from 39.70 to 240.07 May. According to the phylogenetic relationships, the proteins of duplicated genes are close to each other and have higher sequence similarity; for example, the sequence similarity between SlLAX2 and SlLAX5 was found to be 93.15%. Only in two cases, the similarity between the proteins of duplicated genes was found to be below 50%; the similarity between SlLHT1 and SlLHT10 was 38.84%, and that between SlCAT1 and SlCAT4 was 39.19%. To study the selection pressure among the *SlAAT* duplicated gene pairs, K_a_/K_s_ was calculated (Table 2) and the results revealed that they evolved under purifying selection (K_a_/K_s_ < 1). 

### 2.2. Phylogenetic Analysis and Classification of the SlAATs

**Figure 3:**
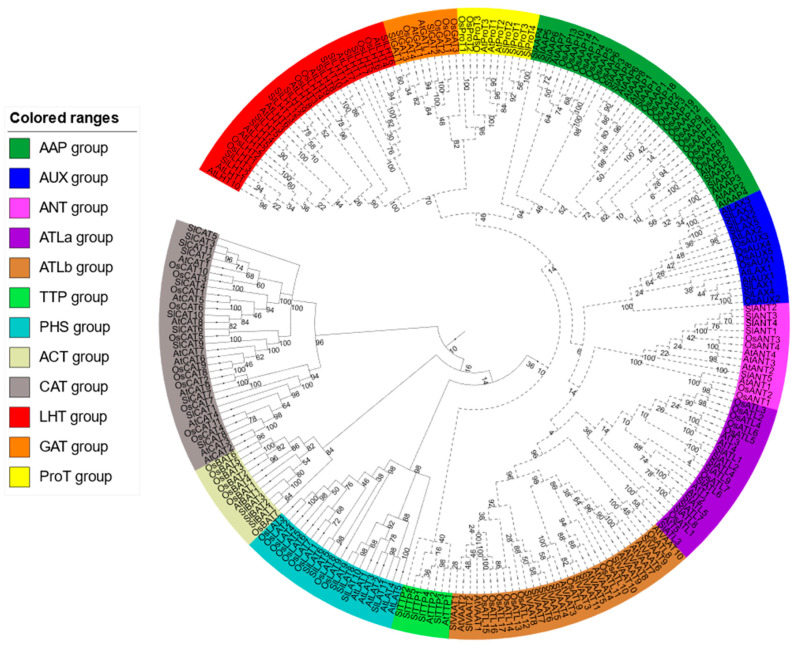
Phylogenetic tree of protein sequences of the AATs of *Solanum lycopersicum*, *Oryza sativa*, and *Arabidopsis thaliana* generated using MEGA 6.0 software with the Maximum Likelihood method and Whelan and Goldman model [35]. The bootstrap consensus tree inferred from 500 replicates is taken to represent the evolutionary history of the SlAATs analyzed. The tree was divided into 12 subgroups, marked by different color backgrounds. AAP, amino acid permease; ANT, aromatic and neutral amino acid transporter; ATL, amino acid transporter-like proteins; BAT, bidirectional amino acid transporter; CAT, cationic amino acid transporter; GAT, γ-aminobutyric acid transporter; LAT, L-type amino acid transporter; LAX, like-auxin influx carriers; LHT, lysine/histidine transporter; ProT, proline transporter; TTP, tyrosine-specific transporter; VAAT, vesicular aminergic-associated transporter; JTT, Jones–Taylor–Thornton. Dashed lines: AAAP family, Solid lines: APC family.

The phylogenetic tree, which that was constructed after the alignment of AAT amino acid sequences of *S. lycopersicum*, *O. sativa* and *Arabidopsis thaliana*, revealed that the 88 SlAATs could be clustered into 12 clades (Figure 3). These SlAATs can be classified into two main superfamilies: AAAP and APC. The AAAP family can be classified into eight subfamilies: AAP, LHT, GAT, ProT, LAX, ANT, VAAT, and ATL. On the other hand, the APC family can be classified into four subfamilies according to phylogenetic tree: ACT, TTP, CAT, and PHS.

**Figure 4:**
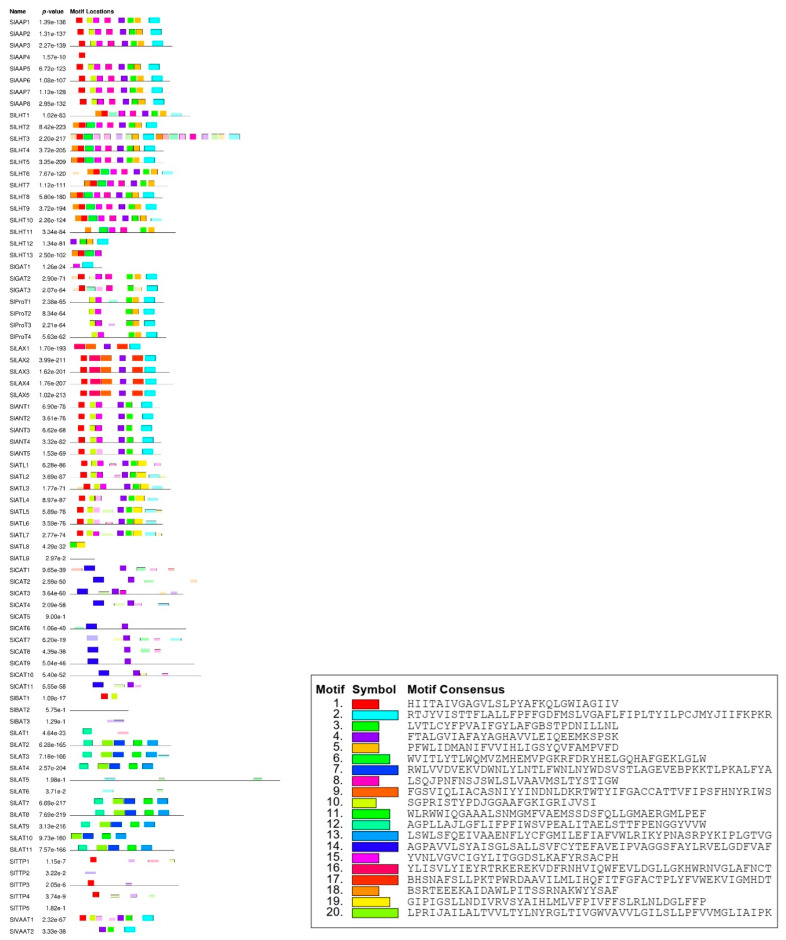
Protein motifs of SlAATs. Each colored box represents a specific motif in the protein identified using the MEME motif search tool (For interpretation of the references to colors in this figure, the reader is referred to the web version of this article.) AAP, amino acid permease; ANT, aromatic and neutral amino acid transporter; ATL, amino acid transporter-like proteins; BAT, bidirectional amino acid transporter; CAT, cationic amino acid transporter; GAT, γ-aminobutyric acid transporter; LAT, L-type amino acid transporter; LAX, like-auxin influx carriers; LHT, lysine/histidine transporter; ProT, proline transporter; TTP, tyrosine-specific transporter; VAAT, vesicular aminergic-associated transporter.

**Figure 5:**
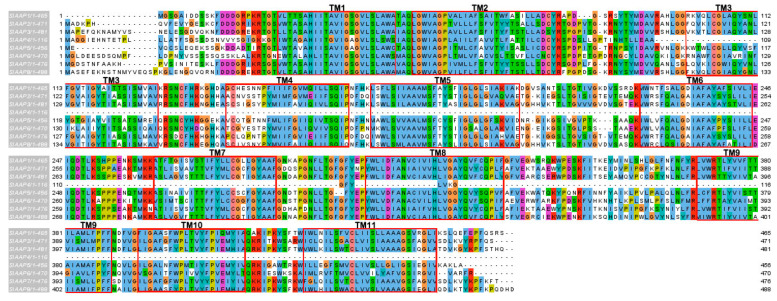
Multiple sequence alignment and TM region of SlAAPs with the default color scheme used for alignments in Clustal X [36]. The TM regions are marked by red. (For interpretation of the references to colors in this figure, the reader is referred to the web version of this article.) TM, transmembrane; AAP, amino acid permease.

Motif analysis using MEME showed that each subfamily has a similar motif and nearly the same their number (Figure 4). For example, all members of the AAP subfamily were found to have the same motifs. In addition, some motifs are more common in one family than the other. For example, motif 1, 2, and 5 are likely to be more common in the AAAP family than the APC family. On the other hand, some motifs were specific to one subfamily; for example, motif 17 was specific to the LAX subfamily and motif 19 was specific to the ATL subfamily. Analysis of the transmembrane region conservation, revealed that most of the transmembrane regions were highly conserved. The alignment of AAP amino acid sequences and the transmembrane conserved region are illustrated as an example in Figure 5. 

### 2.3. Expression Analysis of SlAAT Genes Based on RNA-Seq Data

**Figure 6:**
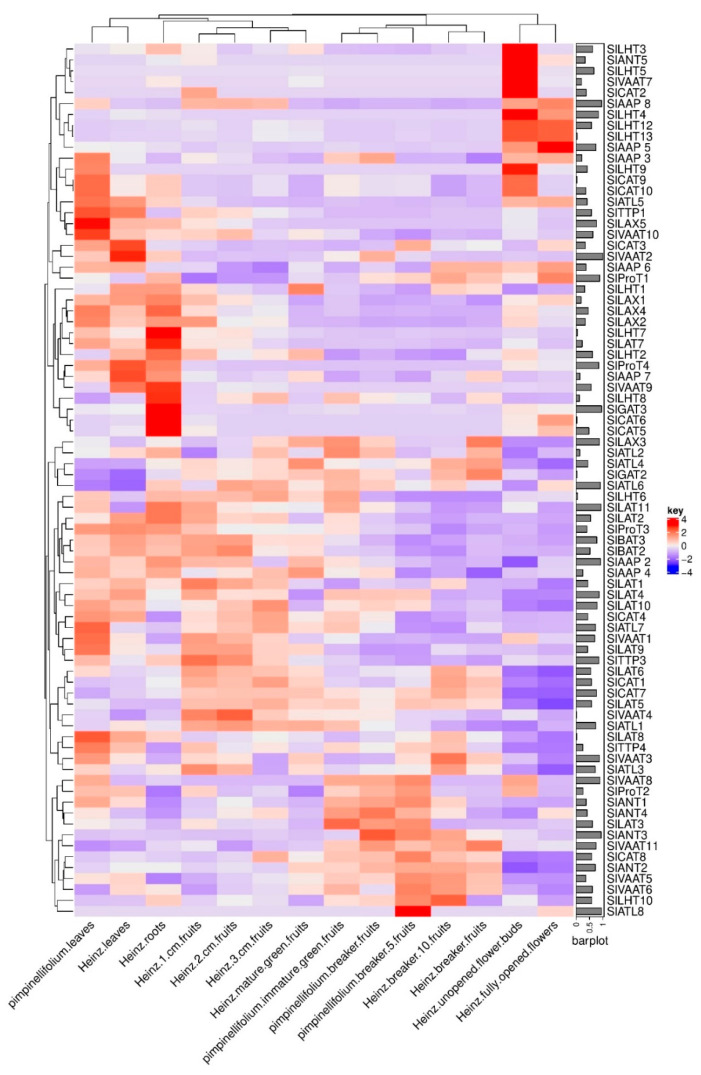
Expression patterns of genes in various tissues of *Solanum lycopersicon* ‘Heinz and their orthologues/paralogs in the wild relative *S. pimpinellifolium*, which were retrieved by the Locus identity number of *SlAATs* included in Table 1. The data for RNA sequencing were obtained from a public database (the Tomato Functional Genomics Database), and were analyzed and generated using ComplexHeatmap version 2.2.0 package in R.

The RNA-Seq data from various tissues during vegetative and reproductive developmental stages of the tomato cultivar *S. lycopersicon* ‘Heinz’ and the wild relative *S. pimpinellifolium* was used to study the expression of *SlATT* genes (Figure 6). According to the column dendrogram, it was observed that there is a similar expression pattern between the leaves of both *pimpinellifolium* and *S. lycopersicon* ‘Heinz, *S. lycopersicon* ‘Heinz 1 cm fruit, *S. lycopersicon* ‘Heinz 2 cm fruit, *S. lycopersicon* ‘Heinz 3 cm fruit, and *S lycopersicon* ‘Heinz mature green fruit, *S. pimpinellifolium* immature green fruit, *S. pimpinellifolium* breaker fruit, *S. lycopersicon* ‘Heinz breaker fruit, and *S. lycopersicon* ‘Heinz breaker^+^ 10 fruit, *S. lycopersicon* ‘Heinz unopened flower buds, and *S. lycopersicon* ‘Heinz fully opened flowers. The expression of *SlLHT3, SlANT5, SlLHT5, SlVAAT7,* and *SlCAT2* was detected to be high only in *S. lycopersicon* ‘Heinz unopened flower buds and extremely low in other examined parts of *S. pimpinellifolium* and *S. lycopersicon* ‘Heinz tomato plants. In addition, six genes, including *SlAAP8, SlLHT4, SlLHT12, SlLHT13, SlAAP5,* and *SlAAP3* showed high expression levels in *S. lycopersicon* ‘Heinz unopened flower buds and fully opened flowers compared to other organs of both *S. pimpinellifolium* and *S. lycopersicon* ‘Heinz tomato plants. 

The expression of *SlLHT9, SlCAT9, SlCAT10,* and *SlATL*5 was considerably high in both *S. lycopersicon* ‘Heinz unopened flower buds and *S. pimpinellifolium* leaves but was low in other examined organs. The expression of *SlTTP1*, *SlLAX5*, *SlVAAT10*, *SlCAT3*, and *SlVAAT2* appeared to be upregulated only in the leaves. Interestingly, *SlVAAT9*, *SlLHT8*, *SlGAT3*, *SlCAT6*, and *SlCAT5* were observed to be highly expressed only in the roots, while the expression in other organs was downregulated. The expression of genes of the AAP family: *SlAAP2*, *SlAAP3*, *SlAAP4*, *SlAAP5*, *SlAAP6*, *SlAAP7*, and *SlAAP8* was observed to be high in some organs such as *S. lycopersicon* ‘Heinz unopened flower buds and *S. lycopersicon* ‘Heinz fully opened flowers, but low in the other examined organs. Six out of 13 genes of the LHT subfamily (*SlLHT1, SlLHT2, SlLHT6, SlLHT7, SlLHT8,* and *SlLHT10*) showed high expression in the roots. The high expression of ProT genes was observed in different organs; for example, *SlProT1* and *SlProT2* were highly expressed in the flowers and in buds respectively, while *SlProT3* and *SlProT4* were highly expressed in both *S. lycopersicon* ‘Heinz leaves and roots. The genes of LAX subfamily were highly expressed in the roots except *SlLAX3,* which showed a high expression in the fruits (*S. lycopersicon* ‘Heinz breaker fruits, *S. pimpinellifolium* breaker fruits, *S. pimpinellifolium* immature green fruits, *S. lycopersicon* ‘Heinz mature green fruits, and *S. lycopersicon* ‘Heinz 3 cm fruits). The expression of *SlBAT2* and *SlBAT3* was the same in all the examined organs. Two genes of the TTP subfamily (*SlTTP1* and *SlTTP4*) exhibited a similar expression pattern: high in the leaves and low in other organs. 

### 2.4. Syntenic Analysis

**Figure 7:**
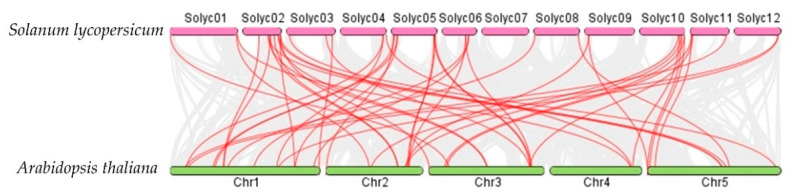
Syntenic analysis between AAt regions of *Solanum lycopersicum* and *Arabidopsis thaliana*. Gray lines in the background indicate collinear blocks between *Solanum lycopersicum* and *Arabidopsis thaliana*, while red lines highlight syntenic AAT gene pairs.

**Figure 8:**
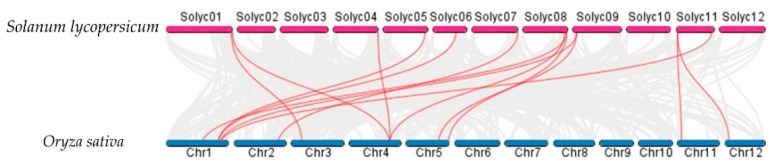
Syntenic analysis between AAt regions of *Solanum lycopersicum* and *Oryza sativa*. Gray lines in the background indicate collinear blocks between *Solanum lycopersicum* and *Oryza sativa*, while red lines highlight syntenic AAT gene pairs.

Syntenic analysis performed among the AATs in *S. lycopersicum*, *Arabidopsis thaliana*, and *O. sativa* demonstrated that the *S. lycopersicum* AATs are orthologs of a number of *O. sativa* and *Arabidopsis thaliana* AAT genes (Figure 7 and Figure 8). For example, *SlLAT5* and *SlLAT6* genes are orthologs of *OsLAT7* (LOC_Os01g61044.1), and *StLAX2* is an ortholog of *OsAUX3* (LOC_Os03g14080), whereas *StLAX4* is an ortholog of *OsAUX1* (LOC_Os01g63770). Syntenic AAT gene pairs between *S. lycopersicum* and *Arabidopsis thaliana*, and between *S. lycopersicum* and *O. sativa* are presented in Supplementary Tables S1 and S2. 

## 3. Discussion

AATS have been identified in several plant species such as *Arabidopsis thaliana*, *O. sativa*, and *S. tuberosum*. However, ATTs in *S. lycopersicum* have not been identified so far. In this study, 88 AATs were identified in *S. lycopersicum* and divided into two superfamilies based on their similarity with previously identified AATs in *Arabidopsis thaliana*, *O. sativa*, and *S. tuberosum* plants [1,5]. Interestingly, the number of members in the AAP subfamily are the same (8 members) as that in *Arabidopsis*, *S. tuberosum*, and *S. lycopersicum* but in *O. sativa*, this number is more than double (19 members). This expansion in the members of AAP subfamily in *O. sativa* may be due to segmental and tandem duplication events [1]. However, the number of members in the same subfamily varies between species. For example, there are six members in the LHT subfamily in *O. sativa*, ten in *Arabidopsis*, 11 in *S. tuberosum*, and 13 in *S. lycopersicum*. In fact, the largest subfamily in *S. lycopersicum* is LHT and the smallest subfamilies are ACT and GAT with only three members each (Table 1). Our results of the phylogenetic analysis were in agreement with those of *O. sativa*, *Arabidopsis*, and *S. tuberosum*. The SlAATs were divided into two main clades: AAAP and APC superfamilies (Figure 3). Similar to *Arabidopsis thaliana*, *O. sativa*, and *S. tuberosum*, the chromosomal mapping of *S. lycopersicum* AAT genes showed that they are distributed throughout the 12 chromosomes, and most *SlAAT* genes were localized on chromosome 2 and the least were found on chromosome 7 (Figure 2). In the current study, we found that segmental gene duplication within the tomato genome mainly contributed to the expansion of SlAAT superfamily in *S. lycopersicum*, and no tandem duplication was found on the gene pairs. This is not the case for *O. sativa*, since segmental and tandem duplication contributes equally to the expansion of the AAT superfamily [1] and in *S. tuberosum*, only tandem duplication contributes greatly to the expansion of the AAT superfamily [5]. Examination of the expression patterns of the segmental duplicated genes of SlAAT superfamily within tomato genome, showed that each gene exhibits a distinct expression pattern. This may suggest that segmental duplication events in the SlAAT superfamily in *S. lycopersicum* do not have overlapping functions and therefore may contribute to the functional divergence of the duplicated genes. 

The analysis of the expression patterns of *SlAAT* genes may provide useful information for determining their function. In the current study, expression analysis of *SlAAT* genes based on RNA-Seq data showed that a number of genes were expressed in specific tissues and developmental stages. As seen in Figure 6, the expression profiles of *SlAAT* genes that were highly expressed may be associated with specific organs, including leaves, root, and flowers, but not fruits. 

The genes encoding SlLAX1, SlLAX2, SlLAX4, and SlLAX5 are specifically expressed in roots. Syntenic analysis revealed that *StLAX1* is an ortholog of *OsAUX1* and *OsAUX2,* which both showed high expression in the roots. In addition, *StLAX1* is an ortholog of *AtAUX1* (AT2G38120), which is known to be primarily expressed in the roots [37]. Further, the expression of *SlLHT2*, *SlCAT5*, and *SlProT4* were observed to be high in the roots. Syntenic analysis showed that *SlLHT2*, *SlAAP2*, and *SlProT2* are orthologs of *AtLHT1*, *AtAAP5*, and *AtProT2*, respectively, which are reported to play a role in amino acid uptake by the roots [20,38,39]. 

In addition, S*lCAT6* expression was high in both *S. lycopersicon* ‘Heinz roots and *S. lycopersicon* ‘Heinz fully opened flowers. *AtCAT6*, was revealed to be involved in amino acid uptake into the sink cells of flowers and root primordia [40]. The expression of *SlAAP8*, the ortholog of *AtAAP6*, was high in *S. lycopersicon* ‘Heinz unopened flower buds and *S. lycopersicon* ‘Heinz fully opened flowers. However, *AtAAP6* expression is mainly present in the xylem parenchyma cells [41]. 

The expression patterns of *SlAAT* genes elucidated in the current study provide foundation for further investigation of the AATs to completely understand their importance and roles in plant physiology and their contribution to plant productivity. Moreover, as AATs are usually induced by abiotic stresses such as salinity and drought [21], it is important to perform further studies on their interaction with the environment to evaluate the plant performance under stress conditions.

## 4. Materials and Methods

### 4.1. Identification of AAT Genes in S. lycopersicum

To identify the AATs in *S. lycopersicum*, those in *Arabidopsis*, *O. sativa*, and *S. tuberosum* [1,5] were used as queries in Basic Local Alignment Search Tool (BLAST) in the SOL Genomics Network (SGN; http://solgenomics.net/ (accessed on 31 January 2021)) against the *S. lycopersicum* genome and protein sequence database (version SL4.0) with default settings. The databases were also queried for homologs using ‘amino acid transporter’ and ‘amino acid permease’ as keywords. The redundant sequences were removed, and the remaining protein sequences were submitted to InterProScan (https://www.ebi.ac.uk/interpro/search/sequence/ (accessed on 31 January 2021)) to scan for AAT domains. Information about gene structure (length, number of introns, length of the open reading frame (ORF), locus accession, amino acid length, and genomic location were all acquired from SGN. Molecular weight (MW) and the theoretical isoelectric point (pI) were predicted using the Compute/Mw tool (http://web.expasy.org/ (accessed on 31 January 2021)). The gene structure of *SlAATs* were also analyzed using Gene Structure Display Server (GSDS) (http://gsds.gao-lab.org/index.php (accessed on 31 January 2021)) [42]. The TMHMM Server version 2.0 (http://www.cbs.dtu.dk/services/TMHMM/ (accessed on 31 January 2021)) was used to predict the putative transmembrane regions in each SlAAT protein [43]. In order to display the gene structure of the exons and introns of SlAATs, the genomic and cDNA sequences of each SlAAT identified in this study were retrieved from the SGN and used as queries in the GSDS (http://gsds.cbi.pku.edu.cn/ (accessed on 31 January 2021)).The nomenclature of *S. lycopersicum* AATs was assigned according to chromosome order and considering their phylogenetic relationship. However, SlLax1, SlLax2, SlLax3, SlLax4, and SlLax5 have been previously named [44].

### 4.2. Chromosomal Mapping of SlAAT Genes and Gene Duplication

The approximate locations of *SlAAT* genes were identified using the available information at SGN, and mapped onto the 12 corresponding *S. lycopersicum* chromosomes using MapChart software [45].

The Plant Genome Duplication Database (PGDD) (http://chibba.agtec.uga.edu/duplication (accessed on 31 January 2021)) was used to download the gene pairs of the *S. lycopersicum* genome and the related information about synonymous substitution rate (K_s_) and nonsynonymous substitution rate (K_a_) was also obtained. Genes distributed nearby and separated by five or fewer genes was selected as the criterion for considering tandem duplicates. Divergence time (Mya, million years ag) of the gene pairs was estimated using the following equation: T = Ks/2x (x = 6.56 × 10^−9^), in which x is the mean synonymous substitution rate for tomato [46].

The sequence similarity between the proteins of duplicated genes was calculated using the sequence manipulation suite programs (https://www.bioinformatics.org/sms2/ident_sim.html (accessed on 31 January 2021)).

### 4.3. Syntenic Analysis

A multiple collinear scanning toolkit (MCScanX) [47] was used to perform an examination of syntenic regions between the AATs of *S. lycopersicum, Arabidopsis thaliana*, and *O. sativa* and the result was plotted using Dual Synteny Plotter software [48].

### 4.4. Phylogenetic Analysis and Sequence Alignment

In order to construct the phylogenetic tree, the multiple sequence alignment AAT proteins of *S. lycopersicum*, *O. sativa*, and *Arabidopsis thaliana* was performed using MEGA 6.0 software, with the default settings of MUltiple Sequence Comparison by Log-Expectation (MUSCLE) alignment [49]. The phylogenetic tree was constructed using the maximum likelihood method with the following settings: test of phylogeny: bootstrap method; substitution type: amino acid, rates among sites, uniform rates; missing data treatments: use all sites; ML heuristic method: nearest-neighbor-interchange (NNI); initial tree for ML: make initial automatically tree; branch swap filter: none; number of threads: 3. For maximum likelihood analysis, the best model of protein evolution was determined in MEGA6 using the ‘find best DNA/protein models’ tool. The best model was the ‘Whelan and Goldman’ (WAG) model [35]. The robustness of the analyses was examined using 500 bootstrap replicates [50]. To analyze the transmembrane region conservation, the amino acid sequences of SlAATs were aligned using MUSCLE with default settings in Jalview version 2 [51].

The motifs of SlAAT proteins were identified using the MEME tool (http://meme-suite.org/tools/meme (accessed on 31 January 2021)) with default settings, except that the maximum number of motifs was defined as 20.

### 4.5. Expression Analysis of SlAAT Genes Based on RNA-Seq Data

Expression profiles of the *SlAAT* genes were obtained from RNA sequencing (RNA-Seq) data from the Tomato Functional Genomics Database (http://ted.bti.cornell.edu/ (accessed on 31 January 2021)) using the transcriptomic analysis of various tissues in the *S. lycopersicon* ‘Heinz and the wild relative *S. pimpinellifolium.* The search was performed using the locus name as a query. The heatmap was generated using ComplexHeatmap version 2.2.0 package in R [52].

## 5. Conclusions

In this study, the AAT gene superfamily in *S. lycopersicum* was identified and analyzed using a broad range of bioinformatic tools. The SGN was first used to retrieve the nucleotide and amino acid sequences of the SlAATs to obtain general information and sequence characterization of SlAATs. Chromosomal localization and gene duplication events in SlAATs were demonstrated. In addition, the phylogenetic relationships and protein motifs of SlAAts were determined. The expression profiles of *SlAAT* genes in various tissues of *S. lycopersicum* were elucidated using RNA-Seq data in the Tomato Functional Genomics Database. This study will facilitate further investigation of the SlAAT gene superfamily and the functional characterization of the members of SlAATs. Further analysis of expression profiles under abiotic stress conditions will reveal their potential roles in response to environmental stress.

## Figures and Tables

**Table 1:** The general information and sequence characterization of 88 *SlAAT* genes.

Gene *^a^*	Locus *^b^*	Gene Structure		ORF (bp) *^e^*	Protein *^f^*			TM^g^	Genomic Location
		Length (bp) *^c^*	Intron *^d^*		Size (aa)	MW (d)	pI		
AAP group									
*SlAAP1*	Solyc01g106800	4015	6	1704	465	51,289.80	9.12	10	ch01:94562712..94558698
*SlAAP2*	Solyc06g060110	2616	5	2025	471	51,696.38	8.22	10	ch06:38031501..38033508
*SlAAP3*	Solyc11g005070	1817	5	1431	476	52,595.65	8.81	11	ch11:61813..63629
*SlAAP4*	Solyc04g077050	4670	6	1931	481	52,581.07	8.66	10	ch04:62016870..62021539
*SlAAP5*	Solyc07g066000	3743	3	1435	116	11,948.89	5.77	2	ch07:67550704..67554446
*SlAAP6*	Solyc07g066010	2863	6	1788	456	50,495.35	8.78	10	ch07:67553145..67556007
*SlAAP7*	Solyc07g066020	4307	6	1624	470	51,989.10	9.02	11	ch07:67557049..67561355
*SlAAP8*	Solyc12g088190	6729	6	1467	488	54,251.36	8.84	9	ch12:63632109..63638837
LHT group									
*SlLHT1*	Solyc01g111980	2796	4	2085	566	61,689.61	9.33	9	ch01:98115988..98118783
*SlLHT2*	Solyc02g093860	3043	7	1612	449	50,376.26	8.75	9	ch02:54561545..54564587
*SlLHT3*	Solyc02g093870	5720	13	2525	838	94,214.15	8.68	18	ch02:54566638..54572357
*SlLHT4*	Solyc03g111040	2349	6	1323	440	49,255.26	9.49	9	ch03:61718834..61721182
*SlLHT5*	Solyc04g011590	3570	7	1609	439	49,164.78	9.13	9	ch04:4020984..4024553
*SlLHT6*	Solyc04g079560	5000	4	2134	525	57,829.74	9.40	9	ch04:63983135..63988134
*SlLHT7*	Solyc04g082220	2368	5	1377	458	50,211.22	8.74	10	ch04:65965546..65967913
*SlLHT8*	Solyc05g009700	3603	7	1634	435	48,676.17	8.96	8	ch05:3896183..3899785
*SlLHT9*	Solyc05g014530	2694	5	1344	447	50,114.23	9.28	10	ch05:8389595..8392288
*SlLHT10*	Solyc10g055740	3459	4	1416	471	52,538.13	9.13	11	ch10:57311236..57314694
*SlLHT11*	Solyc10g055750	4197	4	1491	496	55,434.48	8.66	8	ch10:57325181..57329377
*SlLHT12*	Solyc12g027880	1027	4	663	220	24,854.86	9.34	5	ch12:27932977..27934003
*SlLHT13*	Solyc12g027890	720	1	603	200	22,806.75	5.83	2	ch12:27934068..27934787
GAT group									
*SlGAT1*	Solyc03g071840	744	1	456	151	17,089.44	9.84	3	ch03:18908947..18909690
*SlGAT2*	Solyc08g082080	5806	6	1699	454	50,022.08	8.68	10	ch08:64977392..64983197
*SlGAT3*	Solyc11g066800	6499	6	1365	454	49,886.57	8.98	10	ch11:52596193..52602691
ProT group									
*SlProT1*	Solyc03g096390	4660	6	1828	441	48,541.10	9.34	10	ch03:58462282..58466941
*SlProT2*	Solyc03g096380	8997	6	1642	439	48,640.15	9.14	11	ch03:58427179..58436175
*SlProT3*	Solyc05g052820	4260	6	1530	441	48,418.75	9.25	10	ch05:62989652..62993911
*SlProT4*	Solyc05g052830	6054	6	1473	452	49,478.00	9.33	11	ch05:62996789..63002842
AUX/LAX group									
*SlLAX1*	Solyc09g014380	3773	6	1862	411	46,352.53	9.19	10	ch09:6008319..6012091
*SlLAX2*	Solyc01g111310	3542	7	1838	494	55,728.03	8.86	10	ch01:97592414..97595955
*SlLAX3*	Solyc11g013310	2400	NA	1727	470	53,305.45	9.08	10	ch01:29201232..29203317
*SlLAX4*	Solyc10g076790	4547	NA	1458	485	54,557.53	7.62	10	ch10:59744696..59751060
SlLAX5	Solyc10g055260	4758	NA	1473	490	55,344.70	8.88	10	ch10:56522860..56529521
ANT group									
*SlANT1*	Solyc00g007130	1500	1	1500	421	46,305.95	8.51	11	ch02:6739403..6740902
*SlANT2*	Solyc02g082510	1278	1	1278	425	46,696.39	7.54	11	ch02:46198820..46200097
*SlANT3*	Solyc02g082520	1260	1	1260	420	45,887.14	5.25	9	ch02:46200905..46202164
*SlANT4*	Solyc03g032090	1284	1	1284	427	46,877.54	7.54	11	ch03:4609738..4611021
*SlANT5*	Solyc10g048180	1284	1	1284	427	46,395.87	5.43	11	ch10:44437584..44438867
ATLa group									
*SlATL1*	Solyc02g065680	3430	5	1721	463	50,340.18	8.17	11	ch02:36842710..36846139
*SlATL2*	Solyc02g089400	4076	4	1794	460	49,949.96	8.14	11	ch02:51230438..51234513
*SlATL3*	Solyc03g117350	1419	1	1419	472	51,228.09	5.35	11	ch03:66495836..66497254
*SlATL4*	Solyc04g077460	4788	1	1602	451	49,071.42	7.13	11	ch04:62369207..62373994
*SlATL5*	Solyc05g052300	2152	4	1343	438	48,192.59	7.63	11	ch05:62572297..62574448
*SlATL6*	Solyc06g050790	3039	4	1647	435	47,661.31	5.91	11	ch06:33581820..33584858
*SlATL7*	Solyc06g050800	1895	4	1443	438	47,942.31	8.54	11	ch06:33590884..33592778
*SlATL8*	Solyc09g059850	946	1	363	120	13,678.25	4.19	4	ch09:56191346..56192291
*SlATL9*	Solyc09g059880	554	2	351	116	12,971.29	6.22	3	ch09:56307952..56308505
ATLb group									
*SlVAAT1*	Solyc03g013160	2247	2	1393	426	47,036.67	8.34	11	ch03:47050218..47052464
*SlVAAT2*	Solyc03g013440	4158	3	1183	346	38,031.13	8.44	8	ch03:44654581..44658738
*SlVAAT3*	Solyc03g063030	3167	6	1168	235	25,699.16	4.69	5	ch03:33033162..33036328
*SlVAAT4*	Solyc03g078150	7294	11	2177	525	57,412.83	4.89	10	ch03:50131086..50138379
*SlVAAT5*	Solyc05g050440	615	1	560	171	18,934.73	5.28	5	ch05:60609933..60610547
*SlVAAT6*	Solyc05g053970	1691	1	1186	352	38,856.00	9.12	10	ch05:63976214..63977904
*SlVAAT7*	Solyc06g061260	582	NA	582	193	21,758.22	9.56	4	ch06:39263684..39264265
*SlVAAT8*	Solyc06g061270	2147	1	630	210	23,054.88	7.58	5	ch06:39266520..39268666
*SlVAAT9*	Solyc09g098380	1687	2	1329	442	48,416.47	8.79	11	ch09:72214957.72216643
*SlVAAT10*	Solyc10g084830	4031	10	1590	529	57,492.88	5.05	10	ch10:64225452..64229482
*SlVAAT11*	Solyc11g008440	3427	10	1668	555	60,735.13	5.83	9	ch11:2646308..2649734
CAT group									
*SlCAT1*	Solyc02g037510	16,322	14	2212	598	63,275.37	6.45	15	ch02:30972606..30988927
*SlCAT2*	Solyc02g070270	1884	1	1791	596	64,632.50	8.15	14	ch02:40050344..40052227
*SlCAT3*	Solyc02g070280	4697	2	1991	532	57,650.94	8.88	14	ch02:40054985..40059681
*SlCAT4*	Solyc02g081850	1961	1	1618	483	52,994.82	8.54	10	ch02:45639037..45640997
*SlCAT5*	Solyc08g077810	1575	1	687	228	26,105.16	9.03	5	ch08:61724201..61725775
*SlCAT6*	Solyc08g077820	2804	4	1797	546	60,681.69	8.72	11	ch08:61726678..61729481
*SlCAT7*	Solyc10g018600	9228	7	1710	569	60,434.61	6.34	13	ch10:8819787..8829014
*SlCAT8*	Solyc10g081460	7801	13	1953	650	67,933.20	6.58	14	ch10:62511363..62519163
*SlCAT9*	Solyc11g006710	3720	4	1755	584	63,360.16	7.02	15	ch11:1308228..1311947
*SlCAT10*	Solyc12g011370	1854	NA	1854	617	67,422.93	8.84	13	ch12:4203735..4205588
*SlCAT11*	Solyc12g096380	2356	2	1800	599	65,926.19	8.64	14	ch12:65323438..65325793
ACT group									
*SlBAT1*	Solyc03g063050	2544	3	801	266	29,148.75	6.02	5	ch03:33065762..33068305
*SlBAT2*	Solyc05g008760	2766	6	1407	275	29,637.36	7.71	6	ch05:2991698..2994463
*SlBAT* *3*	Solyc05g008770	7724	7	1051	321	35,247.12	5.28	4	ch05:2993981..300170
PHS group									
*SlLAT1*	Solyc01g005900	3862	2	840	274	30,206.82	6.18	2	ch01:590446..594307
*SlLAT2*	Solyc01g005920	1719	NA	1719	475	52,971.87	8.15	3	ch01:600898..602616
*SlLAT3*	Solyc01g034080	1413	NA	1413	470	52,665.67	8.76	10	ch01:35838791..35840203
*SlLAT4*	Solyc01g111800	1623	NA	1623	461	51,325.78	6.00	9	ch01:97973675..97975297
*SlLAT5*	Solyc02g021620	21,558	12	3376	988	107,982.65	7.11	11	ch02:23288582..23310139
*SlLAT6*	Solyc02g070290	16,229	12	3251	979	107,388.67	5.54	11	ch02:40062787..40079015
*SlLAT7*	Solyc08g005540	4971	1	1593	530	58,240.50	7.64	12	ch08:397461..402431
*SlLAT8*	Solyc08g075710	6457	2	1872	534	58,793.38	8.56	10	ch08:59838882..59845338
*SlLAT9*	Solyc08g078100	1407	NA	1407	468	51,710.24	5.81	10	ch08:61950808..61952214
*SlLAT10*	Solyc09g092420	1786	1	858	285	32,004.77	8.78	5	ch09:71520932..71522717
*SlLAT11*	Solyc10g049640	1929	1	1473	490	54,492.65	5.46	11	ch10:46312657..46314585
TTP group									
*SlTTP1*	Solyc04g049340	2887	7	1775	505	55,314.72	8.64	11	ch04:41501638..41504524
*SlTTP2*	Solyc07g032290	664	1	564	187	20,294.91	5.84	3	ch07:37107752..37108415
*SlTTP3*	Solyc10g078470	1682	1	1542	513	54,551.23	9.14	11	ch10:60286147..60287828
*SlTTP4*	Solyc11g072170	8482	13	1494	497	53,505.37	6.00	10	ch11:55401477..55409958
*SlTTP5*	Solyc12g027810	267	NA	267	88	9313.27	8.55	2	ch12:27130397..27130663

*^a^* Systematic designation given to *Solanum lycopersicum AATs* in this study. *^b^* Locus identity number of *SlAATs* assigned by SGN. *^c^* Full length of *SlAAT* genes obtained from SGN. *^d^* Number of introns in *SlAAT* genes obtained from SGN. *^e^* Length of the open reading frame of the *SlAAT* genes. *^f^* Protein characterization of SlAATs: size obtained from SGN, pI, and MW obtained from ExPASy. *^g^* Number of TM segments possessed by SlAAT proteins, predicted by the TMHMM Server version 2.0. AAT, amino acid transporter; ORF, open reading frame; bp, base pair; aa, amino acids; MW, molecular weight; pI, isoelectric point; TM, transmembrane; NA, not available; Sol Genomics Network, SGN.

**Table 2:** The gene duplication data obtained from the Plant Genome Duplication Database (PGDD).

Duplicated Gene Pairs	K_a_	K_s_	K_a_/K_s_	Time (Mya *)	Percent Similarity %
*SlAAP1/SlAAP4*	0.2425	1.6212	0.149581	123.60	78.47
*SlAAP4/SlAAP8*	0.1367	0.9432	0.144932	71.90	87.50
*SlLHT1/SlLHT7*	0.4291	1.5858	0.270589	120.70	59.44
*SlLHT1/SlLHT10*	0.4985	2.2304	0.223503	170	38.84
*SlLHT2/SlLHT9*	0.2663	2.0484	0.130004	156.13	78.98
*SlProT2/SlProT3*	0.1932	0.6976	0.27695	53.17	83.03
*SlLAX1/SlLAX4*	0.051	0.8422	0.060556	64.19	80.37
*SlLAX2/SlLAX5*	0.0489	0.9088	0.053807	69.27	93.15
*SlANT1/SlANT2*	0.1376	0.6057	0.227175	46.17	85.88
*SlANT2/SlANT4*	0.1182	0.769	0.153706	58.63	88.29
*SlATL1/SlATL2*	0.0807	0.5208	0.154954	39.70	89.85
*SlATL5/SlATL6*	0.3282	1.949	0.168394	148.55	71.62
*SlCAT1/ SlCAT4*	0.8015	3.1497	0.254469	240.07	39.19
*SlLAT2/ SlLAT3*	0.1591	0.9493	0.167597	72.36	86.97
*SlLAT5/ SlLAT6*	0.0685	0.6099	0.112313	46.49	91.33
*SlLAT7/ SlLAT8*	0.1438	0.7838	0.183465	59.740	86.03

Mya, million years ago.

## Data Availability

Not applicable.

## Supplementary Materials

The following are available online at https://www.mdpi.com/2223-7747/10/2/289/s1, Table S1: Syntenic AAT gene pairs between *Solanum lycopersicum* and *Arabidopsis thaliana.* Table S2: Syntenic AAT gene pairs between *Solanum lycopersicum* and* Oryza sativa*.
