# Supplementary material for: Genome Wide Analysis of Amino Acid Transporter Superfamily in Solanum lycopersicum
Source: Plants (Basel). 2021 Feb 3;10(2):289. doi: 10.3390/plants10020289 (PMC7913553; doi:10.3390/plants10020289)
Supplement: Supplementary file 1 [file plants-10-00289-s001.pdf]

plants

MDPI

Table S1: Syntenic AAT gene pairs between *Solanum lycopersicum* and *Arabidopsis thaliana*

Locus[a] (gene name[b])	Locus[c] (gene name[d])
Solyc01g005900 (*SlLAT1*)	AT3G13620 (*AtLAT5*)
Solyc01g111310 (*SlLAX2*)	AT2G21050 (*AtLAX2*)
Solyc01g111980 (*SlLHT1*)	AT4G35180 (*AtLHT7*)
Solyc02g065680 (*SlATL1*)	AT3G30390 (*AtT3*)
Solyc02g065680 (*SlT1*)	AT5G38820 (*AtT4*)
Solyc02g070270 (*SlCAT2*)	AT2G34960 (*AtCAT5*)
Solyc02g081850 (*SlCAT4*)	AT2G34960 (*AtCAT5*)
Solyc02g089400 (*SlATL2*)	AT3G30390 (*AtT3*)
Solyc02g089400 (*SlATL2*)	AT5G38820 (*AtT4*)
Solyc02g093860 (*SlLHT2*)	AT1G67640 (*AtLHT5*)
Solyc02g093860 (*SlLHT2*)	AT3G01760 (*AtLHT6*)
Solyc02g093860 (*SlLHT2*)	AT5G40780 (*AtLHT1*)
Solyc03g032090 (*SlANT4*)	AT5G65990 (*AtANT2*)
Solyc03g096380 (*SlProT2*)	AT3G55740 (*AtProT2*)
Solyc03g117350 (*SlATL3*)	AT1G80510 (*AtT5*)
Solyc04g077050 (*SlAAP4*)	AT1G10010 (*AtAAP8*)
Solyc04g077050 (*SlAAP4*)	AT1G58360 (*AtAAP1*)
Solyc04g082220 (*SlLHT7*)	AT4G35180 (*AtLHT7*)
Solyc05g008760 (*SlBAT2*)	AT2G01170 (*AtBAT1*)
Solyc05g009700 (*SlLHT8*)	AT1G25530 (*AtLHT9*)
Solyc05g014530 (*SlLHT9*)	AT1G67640 (*AtLHT5*)
Solyc05g014530 (*SlLHT9*)	AT1G24400 (*AtLHT2*)
Solyc05g052300 (*SlATL5*)	AT2G40420 (*AtT1*)
Solyc05g052300 (*SlATL5*)	AT3G56200 (*AtT2*)
Solyc05g052820 (*SlProT3*)	AT2G39890 (*AtProT1*)
Solyc05g052820 (*SlProT3*)	AT3G55740 (*AtProT2*)
Solyc06g050790 (*SlATL6*)	AT2G40420 (*AtT1*)
Solyc06g050790 (*SlATL6*)	AT3G56200 (*AtT2*)
Solyc06g060110 (*SlAAP2*)	AT1G77380 (*AtAAP3*)
Solyc06g060110 (*SlAAP2*)	AT1G44100 (*AtAAP5*)
Solyc08g005540 (*SlLAT7*)	AT1G31820 (*AtLAT2*)
Solyc08g082080 (*SlGAT2*)	AT5G41800 (*AtGATL1*)
Solyc09g014380 (*SlLAX1*)	AT2G38120 (*AtAUX1*)
Solyc09g014380 (*SlLAX1*)	AT5G01240 (*AtLAX1*)
Solyc10g055260 (*SlLAX5*)	AT2G21050 (*AtLAX2*)
Solyc10g055740 (*SlLHT10*)	AT4G35180 (*AtLHT7*)
Solyc10g076790 (*SlLAX4*)	AT2G38120 (*AtAUX1*)
Solyc10g076790 (*SlLAX4*)	AT5G01240 (*AtLAX1*)
Solyc10g084830 (*SlVAAT10*)	AT3G09330 (*AtVAAT6*)
Solyc10g084830 (*SlVAAT10*)	AT5G02170 (*AtVAAT8*)
Solyc11g006710 (*SlCAT9*)	AT3G10600 (*AtCAT7*)
Solyc11g006710 (*SlCAT9*)	AT5G04770 (*AtCAT6*)
Solyc11g008440 (*SlVAAT11*)	AT3G54830 (*AtVAAT5*)

Solyc11g066800 (*SlGAT3*)	AT1G08230 (*AtGAT1*)
Solyc12g088190 (*SlAAP8*)	AT1G10010 (*AtAAP8*)
Solyc12g088190 (*SlAAP8*)	AT5G49630 (*AtAAP6*)

[a] Locus identity number of *SlAATs* assigned by SGN.
[b] Systematic designation given to *Solanum lycopersicum AATs* in this study.
[c] Locus identity number of *AtAATs* assigned by TAIR.
[d] Systematic designation given to *Arabidopsis thalian AATs* in [1].
AAT, amino acid transporter; Sol Genomics Network, SGN; The Arabidopsis Information Resource, TAIR.

Table S2: Syntenic AAT gene pairs between *Solanum lycopersicum* and *Oryza sativa*

Locus[a] (gene name[b])	Locus[c] (gene name[d])
Solyc01g111310 (*SlLAX2*)	LOC_Os03g14080 (*OsAUX3*)
Solyc01g111980 (*SlLHT1*)	LOC_Os04g47420 (*OsLHT5*)
Solyc04g082220 (*SlLHT7*)	LOC_Os04g47420 (*OsLHT5*)
Solyc05g052300 (*SlATL5*)	LOC_Os01g61044 (*OsATL7*)
Solyc06g050790 (*SlATL6*)	LOC_Os01g61044 (*OsATL7*)
Solyc07g066000 (*SlAAP5*)	LOC_Os02g49060 (*OsAAP10*)
Solyc08g082080 (*SlGAT2*)	LOC_Os01g43320 (*OsGAT2*)
Solyc08g077810 (*SlCAT5*)	LOC_Os04g45950 (*OsCAT5*)
Solyc08g082080 (*SlGAT2*)	LOC_Os05g50920 (*OsGAT1*)
Solyc09g014380 (*SlLAX1*)	LOC_Os01g63770 (*OsAUX1*)
Solyc09g014380 (*SlLAX1*)	LOC_Os05g37470 (*OsAUX2*)
Solyc11g066800 (*SlGAT3*)	LOC_Os01g63854 (*OsGAT3*)
Solyc11g006710 (*SlCAT9*)	LOC_Os11g05690 (*OsCAT8*)
Solyc11g006710 (*SlCAT9*)	LOC_Os12g06060 (*OsCAT9*)

[a] Locus identity number of *SlAATs* assigned by SGN.
[b] Systematic designation given to *Solanum lycopersicum AATs* in this study.
[c] Locus identity number of *OsAATs* assigned by RGAP.
[d] Systematic designation given to *Arabidopsis thalian AATs* in [1].
AAT, amino acid transporter; Sol Genomics Network, SGN;
Rice Genome Annotation Project, RGAP.
